# Vector competence is strongly affected by a small deletion or point mutations in bluetongue virus

**DOI:** 10.1186/s13071-019-3722-2

**Published:** 2019-10-11

**Authors:** René G. P. van Gennip, Barbara S. Drolet, Paula Rozo Lopez, Ashley J. C. Roost, Jan Boonstra, Piet A. van Rijn

**Affiliations:** 1 Department of Virology, Wageningen Bioveterinary Research, Lelystad, The Netherlands; 2 Arthropod-Borne Animal Diseases Research Unit, Centre for Grain and Animal Health Research, USDA-ARS, Manhattan, KS USA; 3 Kansas State University, Manhattan, KS USA; 4 Department of Biochemistry, Centre for Human Metabolomics, North-West University, Potchefstroom, South Africa

**Keywords:** Bluetongue virus, *Culicoides*, Arbovirus, Vector competence, Virus propagation, Feeding model, Midge

## Abstract

**Background:**

Transmission of vector-borne virus by insects is a complex mechanism consisting of many different processes; viremia in the host, uptake, infection and dissemination in the vector, and delivery of virus during blood-feeding leading to infection of the susceptible host. Bluetongue virus (BTV) is the prototype vector-borne orbivirus (family *Reoviridae*). BTV serotypes 1–24 (typical BTVs) are transmitted by competent biting *Culicoides* midges and replicate in mammalian (BSR) and midge (KC) cells. Previously, we showed that genome segment 10 (S10) encoding NS3/NS3a protein is required for virus propagation in midges. BTV serotypes 25–27 (atypical BTVs) do not replicate in KC cells. Several distinct BTV26 genome segments cause this so-called ‘differential virus replication’ *in vitro*.

**Methods:**

Virus strains were generated using reverse genetics and their growth was examined *in vitro*. The midge feeding model has been developed to study infection, replication and disseminations of virus *in vivo*. A laboratory colony of *C. sonorensis*, a known competent BTV vector, was fed or injected with BTV variants and propagation in the midge was examined using PCR testing. Crossing of the midgut infection barrier was examined by separate testing of midge heads and bodies.

**Results:**

A 100 nl blood meal containing ±10^5.3^ TCID_50_/ml of BTV11 which corresponds to ±20 TCID_50_ infected 50% of fully engorged midges, and is named one Midge Alimentary Infective Dose (MAID_50_). BTV11 with a small in-frame deletion in S10 infected blood-fed midge midguts but virus release from the midgut into the haemolymph was blocked. BTV11 with S1[VP1] of BTV26 could be adapted to virus growth in KC cells, and contained mutations subdivided into ‘corrections’ of the chimeric genome constellation and mutations associated with adaptation to KC cells. In particular one amino acid mutation in outer shell protein VP2 overcomes differential virus replication *in vitro* and *in vivo*.

**Conclusion:**

Small changes in NS3/NS3a or in the outer shell protein VP2 strongly affect virus propagation in midges and thus vector competence. Therefore, spread of disease by competent *Culicoides* midges can strongly differ for very closely related viruses.

## Background

Arthropod-borne viruses have a significant social and economic impact on both human and animal health. The majority of emerging and re-emerging infectious diseases are vector-borne and/or zoonotic [1, 2]. Vector competence has been defined as the capacity of insects to transmit virus, and plays a key role in spread of vector-borne diseases. Competence of insect vectors has been determined by factors related to the insect species and environmental conditions but also involves specific interactions between host, vector and pathogen. Regarding virus-vector interactions, several processes can be recognized; uptake of virus by blood-feeding, replication in the insect vector, dissemination to salivary glands, virus release in saliva and delivery by blood-feeding to the susceptible host, and finally, viremia in the host for subsequent uptake by blood-feeding midges.

Bluetongue (BT) is an insect-borne disease of ruminants which is spread by a limited number of species of biting *Culicoides* midges (Diptera: Ceratopogonidae). Historically, BT is caused by 24 serotypes of bluetongue virus (BTV) (genus *Orbivirus*, family *Reoviridae*) [3], and its worldwide spread is limited to local competent *Culicoides* vectors. The most relevant *Culicoides* species regarding spread of disease are *C. imicola* in Africa/western Asia, *C. obsoletus*, *C. imicola*, *C. dewulfi* and *C. pulicaris* in Europe, *C. sonorensis* in North America/Canada, *C. insignis* in South America, and *C. brevitarsis* in eastern Asia and Australia. Other transmission mechanisms such as vertical transmission in animals seems to be more common for cell-culture adapted BTV, like live attenuated vaccine viruses, than for wild type (wt) BTV1-24 [4, 5]. In addition, animal to animal direct contact transmission leading to viremia has been reported in the field as well as in animal trials [6–8].

Feeding of competent *C*. *sonorensis* midges with blood contaminated with wild type BTV11 (wtBTV11) has resulted in infection, replication and dissemination of wtBTV11 in fully engorged midges [9]. BTV without NS3/NS3a expression is named BT Disabled Infectious Single Animal (DISA) vaccine, since bite transmission by midges is blocked [10]. NS3/NS3a of BTV is not essential for virus replication in a mammalian cell line, but culturing in *Culicoides* cells is abolished by lack of virus release [11], here named ‘differential virus replication’ *in vitro*. Furthermore, NS3/NS3a encoded by genome segment S10 is the prototype virus protein involved in differential virus replication *in vivo*, since virus propagation after intrathoracic inoculation of midges is abolished [9].

In the last decade, new BTV serotypes (25–27) have been found in goats showing unique characteristics [12–14] and are named atypical BTVs [15]. BTV25 could not be isolated despite extensive efforts, but has been successfully passaged in goats using BTV25 containing blood for infection [16]. BTV26 and 27 have been isolated in mammalian cells but culturing in *Culicoides* (KC) cells has failed. Animal trials in vector-free conditions showed virus spread by direct contact transmission [17, 18], but vector-borne transmission of atypical BTVs in the field cannot be ruled out. It has been previously shown that VP2, 5, 7 and NS3/NS3a of atypical BTV25 are functional in the backbone of typical BTV [19]. Similarly, all genome segments S1-10 of BTV26 are functional in BTV1 [RSArrrr/01], although BTV1 with S1[VP1], S3[VP3], or the combination of S2[VP2], S6[VP5], and S7[VP7] of BTV26 did not replicate in KC cells [20]. Since some BTV26 genome segments cause ‘differential virus replication *in vitro*’, virus propagation in competent midges of these BTV1/BTV26 reassortants is likely abolished.

To further investigate differential virus replication *in vitro* and *in vivo*, a small in-frame deletion in NS3/NS3a and typical BTV containing S1[VP1] of atypical BTV26 were evaluated for their ability to replicate in mammalian and *Culicoides* cell lines and in *C. sonorensis* midges. Effects of viral genetics on vector competence is discussed.

## Methods

### Cell lines and viruses

BSR cells (a clone of baby hamster kidney cells) [21] were cultured in Dulbecco’s modified Eagle’s medium (DMEM; Invitrogen, Carlsbad, CA, USA) containing 5% foetal bovine serum (FBS), and antibiotics (100 IU/ml Penicillin, 100 μg/ml Streptomycin and 2.5 µg/ml Amphotericin B) at 37 °C. *Culicoides* (KC) cells were grown in modified Schneider’s *Drosophila* medium with 15% heat inactivated FBS, 100 IU/ml penicillin and 100 μg/ml streptomycin at 28 °C [22].

BTV26 [reference collection sample BTV26-KUW2010/12 BHK2 ex animal B3, [23] (http://www.reoviridae.org/dsrna_virus_proteins/) was purchased from The Pirbright Institute, UK]. A virus stock was obtained by one passage on BSR cells at Wageningen Bioveterinary Research (WBVR) and designated BTV26. BTV11 was isolated from the spleen of a white-tailed deer from Texas in 2011, passaged once in embryonated chicken eggs, and four times in BHK21 cells before use in midge feeding/injecting. A virus stock for *in vitro* experiments was obtained by one passage on BSR cells at WBVR, and designated wtBTV11. All other viruses in this study were generated by reverse genetics [24]. These ‘synthetic’ viruses are based on rgBTV1 [25, 26] and rgBTV11 (this study). After virus rescue, virus stocks were obtained by infection of fresh BSR cell monolayers with a multiplicity of infection (MOI) of 0.1, and stored at 4 °C.

### cDNAs of BTV genome segments

Complete genome segments 1 to 10 (S1-S10) of virus backbones BTV1 (accession numbers FJ969719–28) and BTV11 (GenBank: KM580433–442; [27]) were synthesized as cDNAs by Genscript corporation (Piscataway NJ, USA) in appropriate plasmids under control of the T7 promoter and restriction enzyme sites suitable for run-off RNA transcription [25]. In addition, cDNA of S10 of BTV11 (GenBank: KM580440) was synthesized with an in-frame deletion of 72 amino acid (aa) codons, nucleotide positions 124–339, which encompasses Late Domain motif PPXY/PTAP [28] and corresponds to aa positions 35–106 (S10^del^). Similarly, three chimeric cDNAs encoding S1 [VP1; the RNA-dependent RNA polymerase (RdRp)], containing the same BTV11 as above (S1^11^) and BTV26 (GenBank: JN255156.1; [23]) (S1^26^) sequences were designed and purchased. Each chimeric S1 contained one of three defined domains of the RdRp of BTV26 (S1^11/26^) [29] and untranslated regions of BTV11. Defined VP1 domains correspond to: (i) the N-terminal domain (NTD), nucleotide positions 12–1774 (BTV11chim26S1_NTD); (ii) the polymerase domain (PD), nucleotide positions 1775–2668 (BTV11chim26S1_PD); and (iii) the C-terminal domain (CTD), nucleotide positions 2669–3937 (BTV11chim26S1_CTD). Capped RNA run-off transcripts were synthesized and stored as previously described [25].

### Rescue of BTV variants using reverse genetics

Reverse genetics for BTV as used in this study has been described [24]. Briefly, BSR cell monolayers were transfected with plasmids expressing optimized genes of VP1, 3, 4, 6, and NS1 and 2 followed by transfection with 10 capped run-off RNA transcripts in equimolar amounts after 24 h. At 4 h post-RNA transfection, transfection mix was replaced by culture medium, and virus was harvested as described [11]. Modified or exchanged genome segments were confirmed by partial sequencing according to standard procedures.

### Adaptation to KC cells

To increase virus replication in KC cells, rescued BTV on BSR cells were adapted to KC cells by infecting 2 × 10^5^ KC cells per 2 cm^2^ well with 0.1 ml virus stock. Six days post-infection the supernatant was removed and replaced with 0.2 ml Schneider’s complete medium. Cells were scraped from the bottom and resuspended in 25 cm^2^ flasks with 5 ml Schneider’s complete medium. After 7 days, supernatants were harvested and stored at 4 °C. Cells were scraped from the bottom in 1 ml Schneider’s complete medium and split 1:10 in 5 ml Schneider’s complete medium and grown again for 7 days. The procedure was repeated to generate p2r and p3r. The harvested BTV11(S1^26^) of p3r was designated BTV11(S1^26^)kc(r) and was used to infect fresh KC cell monolayers in 25 cm^2^ flasks at low MOI of 0.1. Supernatants were harvested at 7 days post-infection (dpi) (3pr+p1). Virus passages were repeated, resulting in p3r+3p, here designated BTV11(S1^26^)kc. Passages of KC cells infected with synthetically derived BTV11 (rgBTV11) and BTV11 variants with chimeric VP1 proteins were not needed to harvest virus. Even more, passage of these viruses on KC cell monolayers was successful, whereas infection and subsequent adaptation of BTV26 on KC cell monolayers failed.

### Immunoperoxidase monolayer assay (IPMA)

BTV infection of cell monolayers was confirmed by immunoperoxidase monolayer assay (IPMA) according to standard procedures as previously described for BTV [25]. Briefly, fixed infected monolayers were incubated with monoclonal antibody ATCC-CRL1875 against BTV VP7 followed by conjugated rabbit α-mouse serum (DAKO, Leuven, Belgium).

### Full genome sequencing of BTV11 variants

RNA from different virus stocks was isolated through High Pure viral RNA kit (Roche, Basel, Switzerland) and all ten genome segments were amplified with the OneStep RTPCR kit (Qiagen, Hilden, Germany) using virus specific primers as described [25]. Amplified cDNAs were sequenced using the BigDye® Terminator v3.1 Cycle Sequencing Kit in a ABI PRISM® 3130 Genetic Analyzer (both supplied by Applied Biosystems, Foster City, IA, USA). Sequencing of ultimate 5′- and 3′-ends of genome segments was performed by a modified method. Therefore, infected BSR monolayers were harvested at total cytopathogenic effect (CPE). A volume of 0.1 ml Trizol/cm^2^ monolayer was added and cells were incubated for 5 min at room temperature. After harvesting disrupted cells, 0.2 ml chloroform/ml Trizol was added and the mixture was centrifuged for 10 min at 6200×*g.* The water phase was collected, and 0.8 ml isopropanol/ml was added. Precipitated RNA was centrifuged for 30 min at 4 °C and 13,000× *rpm*. The pellet was washed with 70% ethanol and dissolved in 100 μl RNase-free water. Fifty μl of 7 M LiCl was added, followed by incubation for 30 min at −20 °C to precipitate ssRNA. After centrifugation for 15 min at 4 °C and 13,000× *rpm*, dsRNA was purified from the supernatant using the RNA clean and concentrator^tm^-5 kit (Zymo research, Irvine, CA, USA) according to manufacturer’s protocol. Two-hundred ng anchor oligo PC3-T7loop [30] was ligated to 100 ng dsRNA with T4 RNA ligase (Bioke, Leiden, the Netherlands) according to manufacturer’s conditions for 2 h at 37 °C. RNA was purified using RNA clean and concentrator^tm^-5 kit (Zymo research). Ligated RNA was reverse transcribed using random primers with Superscript III (Invitrogen, Carlsbad, CA, USA) according manufacturer’s conditions, and cDNA was amplified with PC2 [30] and a specific internal primer for each end of each genome segment with TakaraZ Extaq (Takara Bio, Göteborg, Sweden).

### Growth kinetics and virus release

To determine virus replication, monolayers of 2 × 10^5^ BSR cells or 2 × 10^6^ KC cells in 2 cm^2^ wells were infected in duplicate at a multiplicity of infection (MOI) of 0.1. To study virus release, monolayers of 5 × 10^5^ BSR cells or 5 × 10^6^ KC cells in 2 cm^2^ wells were infected with an MOI of 0.01. After virus attachment for 1.5 h at 37 °C to BSR cells, or at 28 °C to KC cells, media was removed and monolayers were washed twice with phosphate-buffered saline (PBS), and 1 ml DMEM complete medium (BSR cells) or 1 ml Schneider’s complete medium (KC cells) was added. This time point was set as 0 h post-infection (hpi). Infected monolayers were incubated at appropriate temperature for indicated hpi, and were subsequently stored at −80 °C. In case of virus release assays, cells and culture medium were separately harvested. Fractions containing cells were lysed by freeze-thawing at −80 °C, centrifuged, and supernatant was stored. Virus titers in each sample were determined by infection of BSR cells with tenfold dilutions. After incubation for 72 h, wells were monitored for CPE and immunostaining by IPMA. Virus titers were expressed as tissue culture infective doses (TCID_50_/ml or log_10_ TCID_50_/ml). Growth kinetics and virus release assays were determined at least twice and virus titrations were independently repeated.

### Feeding and inoculation of midges

Feeding of midges were performed as previously described [9]. For feeding, colonized 3–4 day-old female *C. sonorensis* midges from the Arthropod-Borne Animal Diseases Research Unit, Manhattan, KS, USA [31] were offered a blood meal, consisting of 1:1 (v/v) defibrinated sheep blood and indicated virus titer in an artificial feeder using a parafilm membrane [32]. Midges were allowed to feed for 2 h. Then, they were anesthetized for 10–15 s with CO_2_ and sorted as to blood-feeding status on a CO_2_ fly pad (Diamed Lab Supplies, Inc., Mississauga, Ontario, CA). Twenty-five engorged females were immediately placed in 100 μl RNAlater (Qiagen, Germantown, MD, USA) and stored at 4 °C. This time point was set as 0 days post-feeding (0 dpf). Further, engorged females were put in cardboard cages with cotton plugged vials containing 10% sucrose and held at 26 °C for 10 days (10 dpf). At 10 dpi, 25 midges were decapitated using ultra-fine tweezers (EMS Hatfield, PA, USA) and a dissecting microscope (SMZ 1500; Nikon Instruments, Melville, NY, USA). Heads and bodies were separately placed in 100 μl RNAlater and stored at 4 °C.

For inoculation, colonized 3–4 day-old female *C. sonorensis* midges were intrathoracically microinjected (Nanoject II, Drummond Scientific, Broomall, PA, USA) with 46 nl cell culture media containing indicated virus titer [9]. Twenty-five injected midges were placed in 100 μl RNAlater 1–4 h post-injection, and stored at 4 °C. This time point was set as 0 days post-inoculation (0 dpi). Any variation in time that occurred after injection was due to the time-intensive nature of microinjection of midges. Similar to fed midges, inoculated midges were held at 26 °C for 10 days (10 dpi), and groups of 25 midges were processed as described above.

### RNA isolation and PCR testing

To study the presence of BTV-RNA, bodies and heads were PCR tested as described [9]. Briefly, 400 μl PBS and one 5 mm stainless steel ball (Qiagen) were added to individual bodies and heads in RNAlater in micronic tubes. Tubes were shaken for 3 min at 50 Hz in a tissue lyser (85600, Qiagen). After centrifugation, 200 μl of supernatant was used for RNA isolation. BTV-RNA was detected by the panBTV Seg-10 PCR test or the real time RT-PCR test for Seg-1 [33] adapted to the all-in-one method [33, 34]. Crossing point (Cq) values were calculated, and negative results were arbitrarily set as 45. Due to the maximum of 45 cycles, the highest Cq value that could still be calculated was 40.

## Results

### Rescue of BTV11

**Fig. 1:**
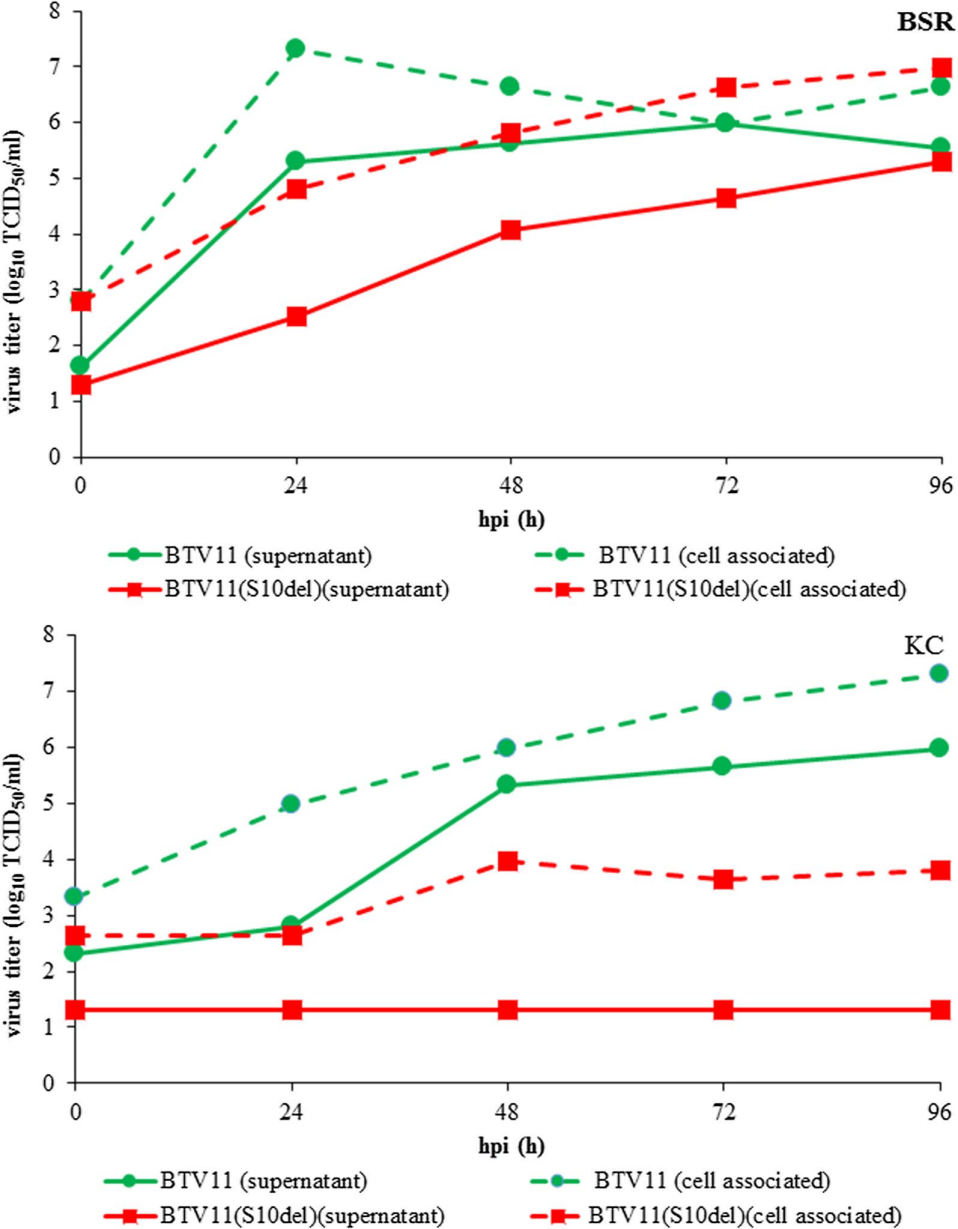
Virus release from BSR and KC cells. Cell-associated virus (dashed lines) and released virus (lines) were determined for BTV11(S10^del^)(squares) and rgBTV11 (circles) in infected monolayers of BSR and KC cells at indicated hours post-infection (hpi). Virus titers are expressed aslog_10_ TCID_50_ per ml. Representative results are shown

To study the role of viral proteins in BTV replication in midges, we first regenerated BTV11 by reverse genetics (rgBTV11). As expected, rgBTV11 efficiently replicated *in vitro* in BSR and KC cells (Fig. 1). We also showed that rgBTV11 replicates in competent midges like wild type BTV11 (wtBTV11) as previously shown [9]. Thus, rgBTV11 is indistinguishable from wtBTV11 and an attractive virus backbone to study the role of viral proteins in differential virus replication *in vitro* and *in vivo*.

### BTV11 with an in-frame deletion in NS3 is not released from KC cells

BTV11 was rescued with Seg-10 encoding NS3/NS3a lacking 72 aa codons from position 36 to 107 of NS3 (S10^del^) encompassing Late Domain. BTV11(S10^del^) developed small plaques (CPE) on BSR monolayers similar to previous NS3 knockout mutants of BTV1, BTV6/net08, and BTV8/net06 [10, 11, 35]. Growth and release of BTV11(S10^del^) was studied in BSR and KC cell cultures (Fig. 1). BTV11(S10^del^) replicated slower in BSR cells than rgBTV11, although both reached a virus titer in the cell-associated fraction and culture medium of ±7 and ±5.5 log_10_ TCID_50_/ml, respectively, at 96 hpi.

In KC cells, BTV11(S10^del^) marginally replicated and stabilized at ±4 log_10_ TCID_50_/ml. In contrast, rgBTV11 steadily grew to ±7 log_10_ TCID_50_/ml at 96 hpi (Fig. 1). Clearly, BTV11(S10^del^) was not released into culture medium of KC cells, whereas rgBTV11 was readily released from KC cells to 5.3 log_10_ TCID_50_/ml at 48 hpi, and further increased to 7.3 log_10_ TCID_50_/ml at 96 hpi. This suggests that the Late Domain of NS3/NS3a protein is essential for virus propagation in KC cells, but not essential for virus replication in BSR cells, which is here named ‘differential virus replication’ *in vitro*.

### Domains in VP1 of BTV26 are not responsible for differential virus replication *in vitro*

BTV1-based reassortants with genome segment 1 of BTV26 (S1^26^) expressing VP1 (RdRp) did not replicate in KC cells [20]. Here, we used this finding to map domains in RdRp involved in differential virus replication *in vitro*. BTV 11/26 chimeric S1 segments encoding one out of three defined RdRp domains of VP1^26^ were incorporated in BTV11 using reverse genetics. All three BTV11 mutants expressing these chimeric VP1^11/26^ proteins with domain NTD, PD or CTD of VP1^26^ were rescued, and could be serially passed in both BSR cells and KC cells. Apparently, none of the RdRp domains of VP1^26^ is involved in differential virus replication. In conclusion, with this approach, we were unable to identify domains in VP1^26^ involved in differential virus replication. We suggested that several domains of VP1^26^ contribute to differential virus replication or that entire VP1^26^ in the BTV11 backbone is functional in virus replication in KC cells.

### BTV11 expressing VP1 of BTV26 replicates in KC cells after adaptation

**Fig. 2:**
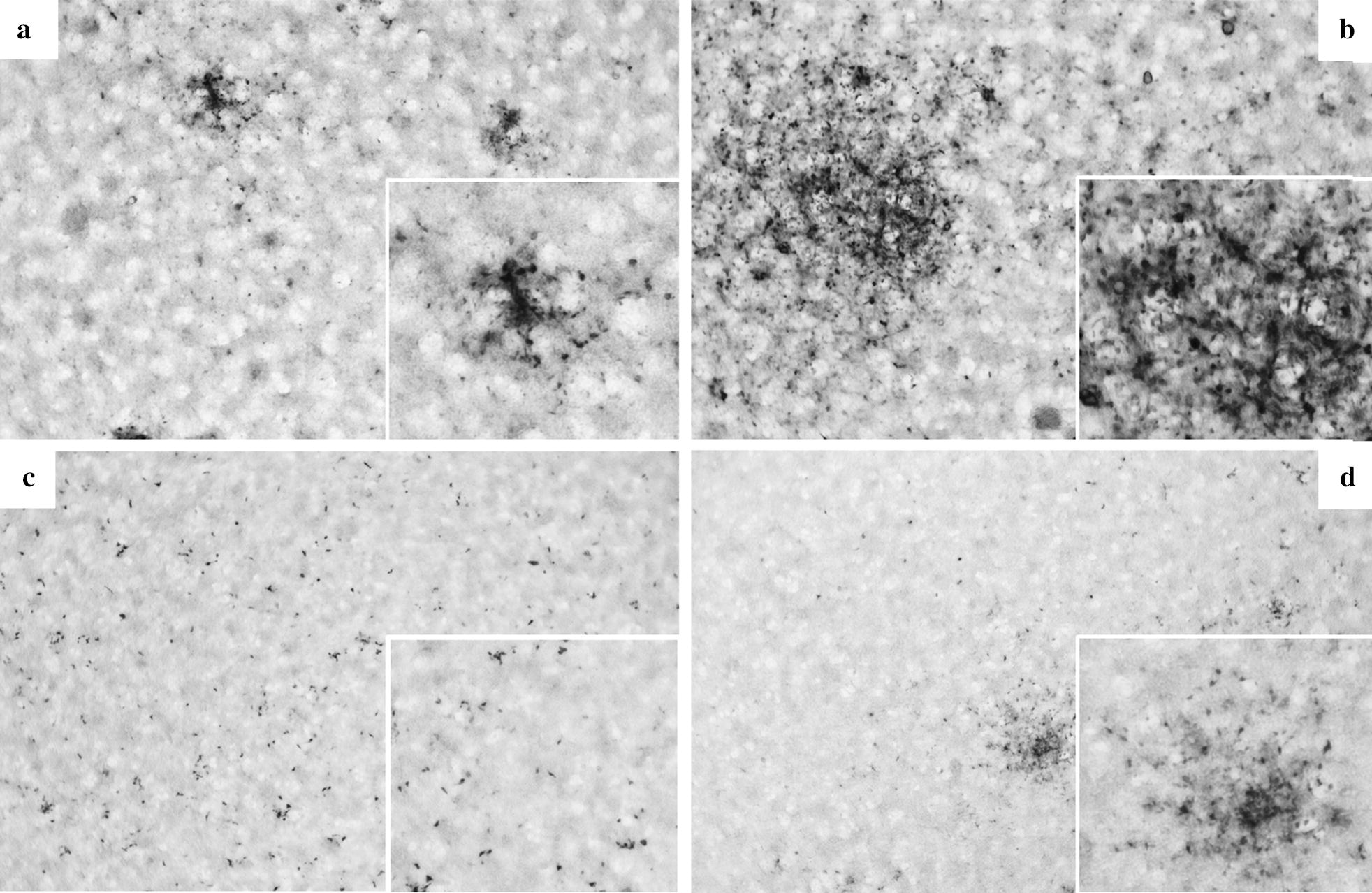
Representative IPMA results. KC monolayers were infected with rgBTV11 or BTV11kc (**a** and **b**), or with BTV11(S1^26^) or BTV11(S1^26^)kc (**c** and **d**). Monolayers were immunostained by IPMA with VP7 specific monoclonal antibody at 3 days post-infection. Clearly, larger immunostained foci were observed for the KC passaged viruses (**a** *versus* **b** and **c** *versus* **d**)

As a next step, BTV11(S1^26^) expressing VP1 of BTV26 was rescued. Rescue of BTV11(S1^26^) was less efficient than its ancestor rgBTV11 or BTV11 expressing VP1^11/26^ protein, as described above. Transfected cells were passed once to obtain cytopathogenic effect (CPE). Harvested BTV11(S1^26^) was passed once on fresh BSR cells and virus stocks were used for subsequent experiments. Initially, BTV11(S1^26^) was not detected in culture medium of infected KC cell monolayers but some KC cells were immunostained (Fig. 2c), suggesting very weak protein expression and possibly virus replication. Duplicate infected KC cell monolayers were blindly passaged three times to ‛rescue’ virus. Virus was harvested from the 3rd passage (p3r) and was named BTV11(S1)^26^kc(r). This virus was subsequently passed three times by infection of fresh KC cell monolayers (p3r+p3), and was named BTV11(S1^26^)kc. In parallel, BTV11(S1^26^) was passaged three times on BSR cells by infection of fresh BSR cell monolayers, and was named BTV11(S1^26^)bsr.

**Fig. 3:**
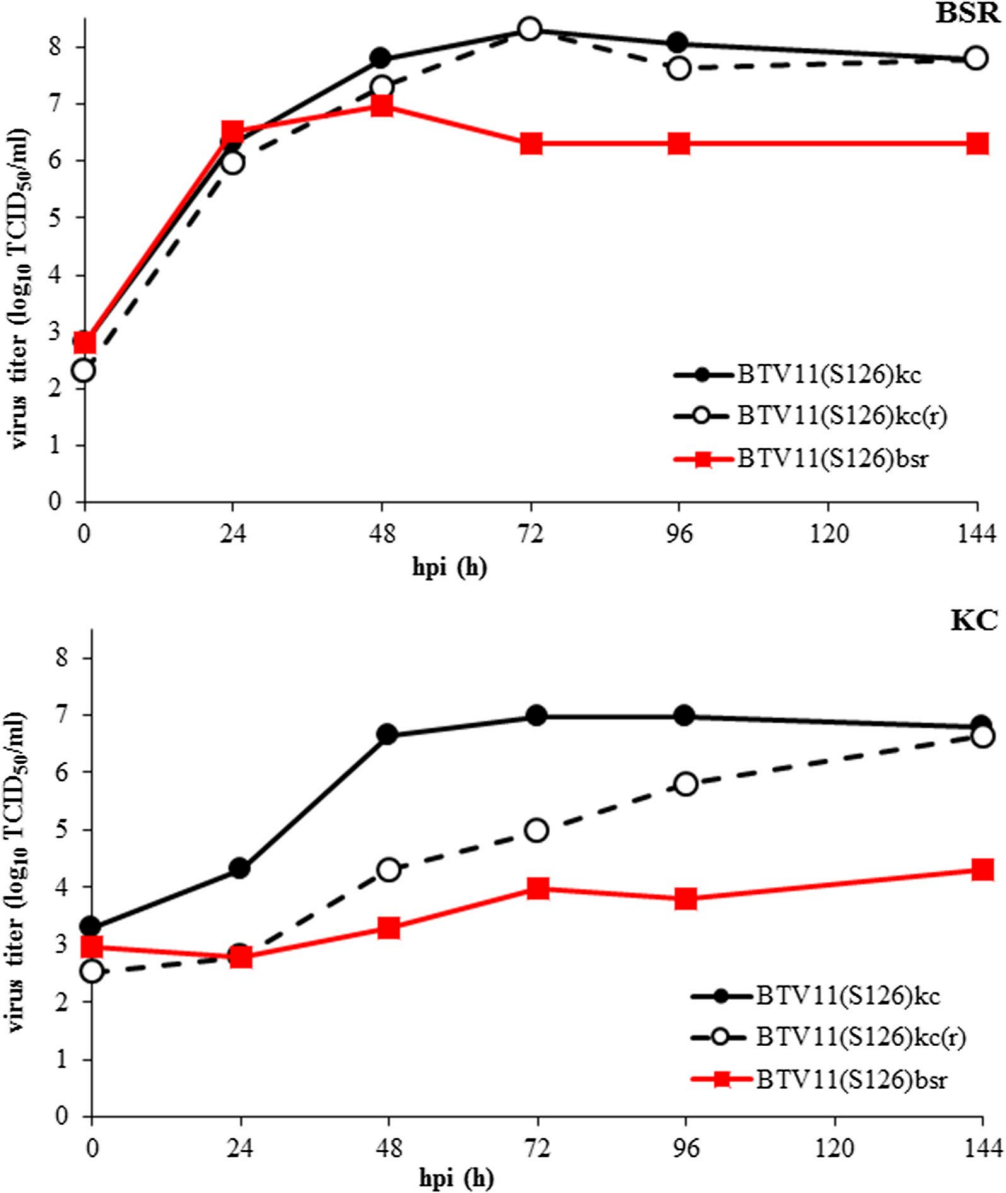
Virus replication of BSR- and KC-adapted BTV11 expressing VP1 of BTV26. Virus replication was studied for BTV11(S1^26^)bsr (squares), BTV11(S1^26^)kc(r) (open circles), and BTV11(S1^26^)kc (filled circles) in BSR and KC cells. Virus titers were determined at indicated hours post-infection (hpi), and expressed as log_10_ of 50% Tissue Culture Infective Dose (TCID_50_) per ml. Representative results are shown

Virus growth of BTV11(S1)^26^kc(r), BTV11(S1^26^)kc and BTV11(S1^26^)bsr was studied in BSR and KC cells (Fig. 3). BTV11(S1^26^)bsr replicated in BSR cells, whereas replication in KC cells was marginal (Fig. 3). Similar results have been obtained with VP1^26^ in the BTV serotype 1 backbone [20]. BTV11(S1^26^)kc(r) and BTV11(S1^26^)kc replicated similar to BTV11(S1^26^)bsr in BSR cells up to 48 hpi but reached higher virus titers at 72 hpi. In contrast to BTV11(S1^26^)bsr, BTV11(S1^26^)kc(r) replicated in KC cells, and was even higher (after three subsequent virus passages) for BTV11(S1^26^)kc. Both KC variants grew to virus titers of ±7 log_10_ TCID_50_/ml at 144 hpi, indicating favouring virus replication in KC cells. Remarkably, BTV11(S1^26^)kc(r) and BTV11(S1^26^)kc also reached higher virus titers on BSR cells than BTV11(S1^26^)bsr. To unravel adaptation mutations in more detail, rgBTV11 was also virus passaged three times on BSR or KC cells resulting in BTV11bsr and BTV11kc, respectively. BTV11(S1^26^)kc and BTV11kc formed larger immunostained foci at 72 hpi than BTV11(S1^26^)bsr and BTV11bsr, respectively (Fig. 2). These results demonstrate that rgBTV11kc was also adapted to virus growth on KC cells.

**Fig. 4:**
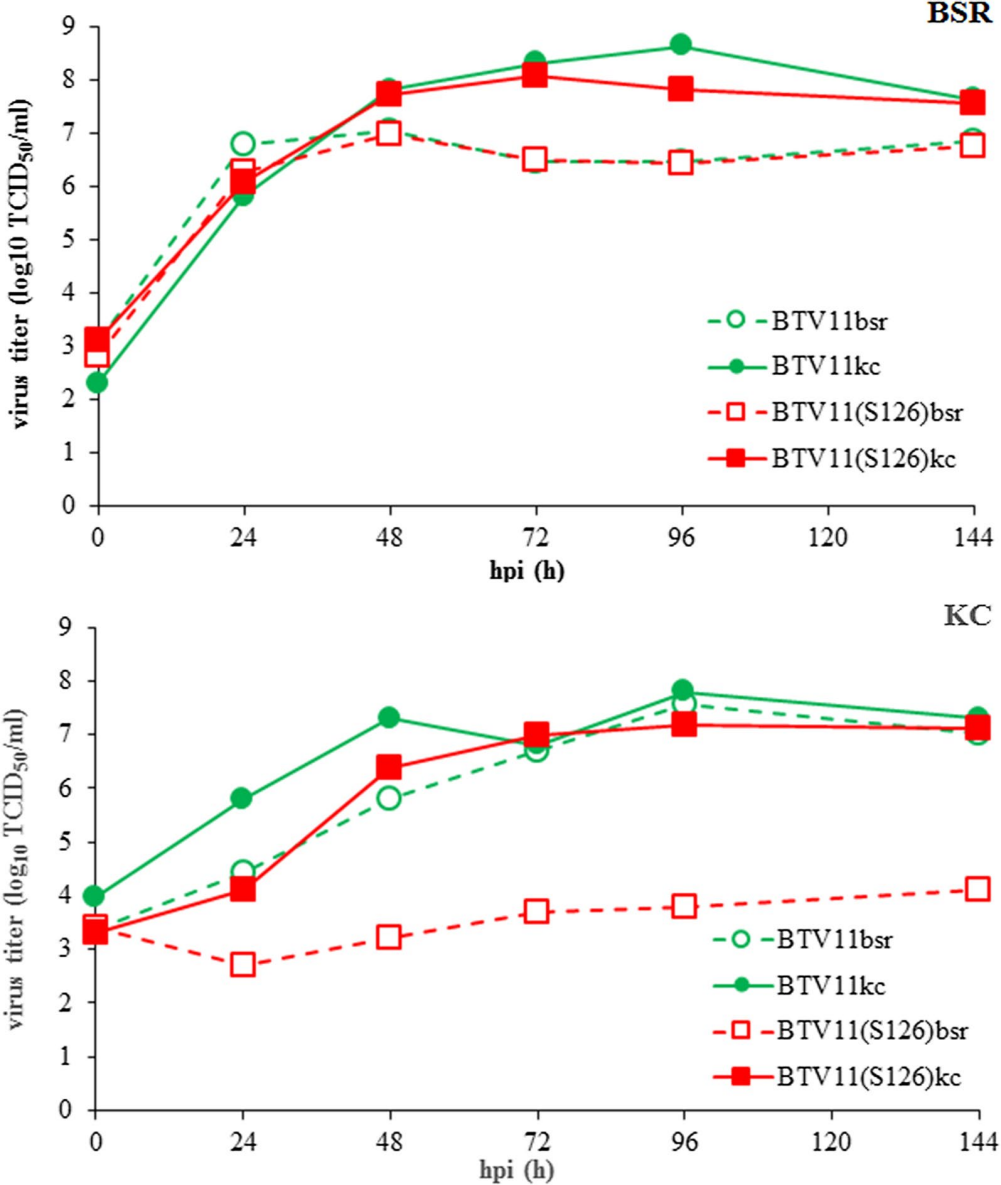
Comparison of virus replication of BTV11 variants after KC adaptation. Virus replication was studied for rgBTV11 and BTV11(S1^26^) after passages in BSR or KC cells as indicated; BTV11bsr (open circle, dashed line), BTV11kc (filled circle, line), BTV11(S1^26^)bsr (open square, dashed line), and BTV11(S1^26^)kc (filled square, line). Virus titers were determined at indicated hours post-infection (hpi), and expressed as log_10_ of 50% Tissue Culture Infective Dose (TCID_50_) per ml. Representative results are shown

Adapted variants of rgBTV11 and BTV11(S1^26^) were studied for virus growth on BSR or KC cells (Fig. 4). Both rgBTV11kc and BTV11(S1^26^)kc replicated to higher virus titers in BSR and KC cells, although the difference in BSR cells was less obvious than in KC cells. Further, we conclude that adaptation to KC cells increased virus replication in both BSR and KC cells, although this difference is less obvious for rgBTV11 than for BTV11(S1^26^).

### Adaptation mutations are subdivided into chimeric corrections and virus growth in KC cells

BTV11(S1^26^)bsr, BTV11(S1^26^)kc(r) and BTV11(S1^26^)kc were completely sequenced and compared to cDNA sequences used to rescue BTV11(S1^26^) (Table 1). BTV11(S1^26^)bsr contained incomplete nucleotide changes in S1[VP1]^26^, S2[VP2]^11^, S4[VP4]^11^, and S8[NS2]^11^. Subsequent virus passages on BSR cells (p6) resulted in three aa mutations; one in S1[VP1]^26^, two in S8[NS2]^11^, and one incomplete aa mutation in S4[VP4]^11^, as well as one incomplete silent mutation in S2[VP2]^11^. These five nucleotide changes were also found after ‘rescue’ of BTV11(S1^26^) in KC cells (p3r) but four aa mutations were not completely changed immediately after ‘rescue’ in BTV11(S1^26^)kc(r). Apparently, these aa mutations were rapidly selected in both cell types and are likely associated with chimeric corrections in BTV11(S1^26^). In addition, one incomplete E321G aa mutation in VP2^11^ was found. After three subsequent virus passages (p3r+3p), BTV11(S1^26^)kc contained three additional incomplete mutations, whereas the incomplete E321G aa change in VP2^11^ was complete (Table 1). Silent mutation A53C in S1[VP1]^26^ was complete after three subsequent virus passages (p3r+p6), whereas two other incomplete changes remained with mixed nucleotide mutations. The respective regions of BTV11kc and BTV11bsr were also sequenced, but no mutations were found, except for aa mutation E321G in S2[VP2]^11^ in BTV11kc. This indicated that the aa mutation E321G in S2[VP2]^11^ is associated with virus growth in KC cells.

**Table 1:** Overview of mutations in BTV11(S1^26^) variants. BTV11(S1^26^) was passed on BSR or KC cells as described. Rescued virus on KC cells (p3r) and variants after three virus passages (p3) on BSR or KC cells were completely sequenced, whereas virus variants after three additional virus passages (p6 and p3r+p6) were partially sequenced to confirm previously observed mutations. Mutations associated with adaptation to KC cells are in bold

Genome segment	BTV11(S126) on BSR cells			BTV11(S126) on KC cells			
	BTV11(S126)bsr (p3)	p6	aa mutation	BTV11(S126)kc(r) (p3r)	BTV11(S126)kc (p3r+p3)	p3r+p6	aa mutation
S1	–	nd	–	–	**A53C** ^a^	**A53C**	**silent**
S1	A937G^a^	A937G	D309G	A937G	A937G	A937G	D309G
S1	–	nd	–	–	**G3287A (minor)** ^**b**^	**G3287A (minor)** ^**b**^	**M1092I**
S2	–	nd	–	**A981G** ^a^	**A981G**	**A981G**	**E321G**
S2	C2392T* (minor)	C2392T(minor)^b^	Silent	C2392T (minor)^b^	C2392T (minor)^b^	C2392T (minor)^b^	Silent
S3	–	nd	–	–	**C2235T(minor)** ^**b**^	**C2235T(minor)** ^**b**^	**R740C**
S4	T1204C^a^	T1204C^a^	L399S	T1204C	T1204C	T1204C	L399S
S5	–	nd	–	–	–	nd	–
S6	–	nd	–	–	–	nd	–
S7	–	nd	–	–	–	nd	–
S8	C530T^a^	C530T	L171F	C530T	C530T	C530T	L171F
S8	A681G^a^	A681G	E221G	A681G	A681G	A681G	E221G
S9	–	nd	–	–	–	nd	–
S10	–	nd	–	–	–	nd	–

^a^ ±50% or mixed nucleotides for each genome segment S1–10

^b^ < 50% or minor nucleotide mutations for each genome segment S1–10

*Notes*: Nucleotide positions are according to positions in the respective genome segments. aa mutations are shown in the right column of each passaged BTV11 variant. Note: mutation A981G in S2[VP2]^11^ resulting in aa mutation E321G was also found for BTV11kc

*Abbreviations*: nd, genome segments of p6 and p3r+p6 without mutations in previous variants were not sequenced; –, no mutations

In summary, a total of nine mutations were found in several genome segments after rescue and passages of BTV11(S1^26^) in KC cells. Seven out of nine mutations resulted in aa mutations. Five point mutations (four aa mutations and one silent mutation) are associated with corrections of chimeric interactions, since in BTV11(S1^26^)bsr contained the same mutation. Four additional point mutations (three aa mutations and one silent mutation) seem to be associated with adaptation to KC cells, although the selection pressure was not very high since two aa mutations were still incomplete after six virus passages in KC cells. The most obvious and strongly selected change is aa mutation E321G in VP2^11^. Even more, rgBTV11 also contained this E321G mutation after virus passages in KC cells.

### Oral infection of midges is dependent on the virus titer in the blood meal

**Fig. 5:**
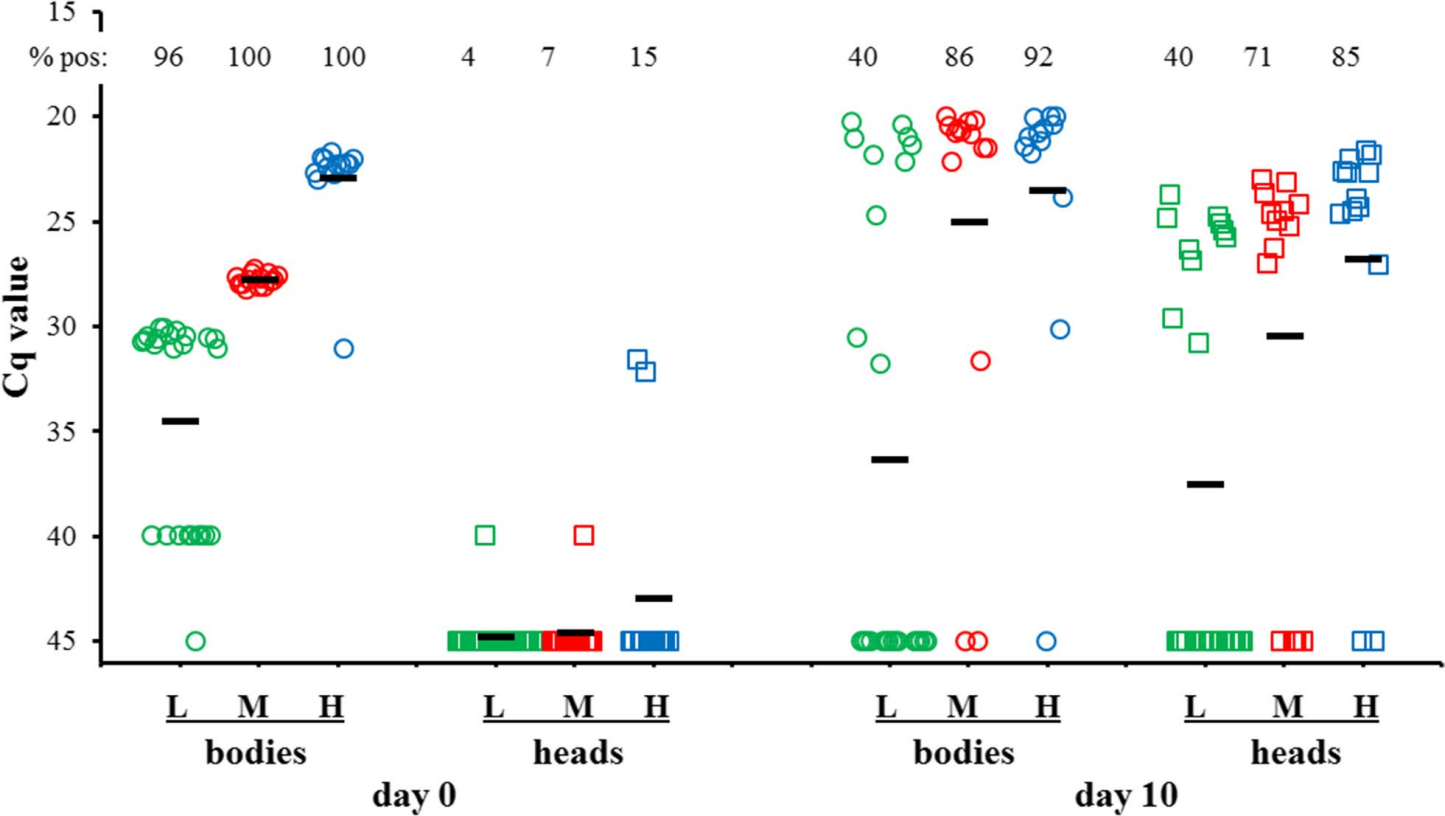
Infection, replication and dissemination of wtBTV11 after feeding with different virus doses. Colonized *C. sonorensis* were fed with blood containing different virus titers of wild type (wt) BTV11, L: 10^5.1^ TCID_50_/ml; M: 10^6.3^ TCID_50_/ml; H: 10^8.2^ TCID_50_/ml. Viral RNA was detected and semi-quantitated by PCR expressed in Cq values for individual bodies (circles) and heads (squares) at 0 and 10 dpf. The mean Cq value (bars) and percentage of PCR-positives of each group are indicated

Virus propagation *in vivo* was studied in blood-fed midges. For this, individual bodies and heads were tested by PCR directly after feeding (0 dpf) or at 10 dpf to distinguish between infection, replication and dissemination of virus [9]. Firstly, we roughly determined the 50% infective virus dose by feeding blood containing different virus titers of wtBTV11; L: 10^5.1^ TCID_50_/ml, M: 10^6.3^ TCID_50_/ml, H: 10^8.2^ TCID_50_/ml. Fully engorged midges were selected and processed at 0 dpf to confirm uptake, and at 10 dpf to study virus replication and dissemination (Fig. 5).

Bodies of all groups were PCR-positive at 0 dpf, except for one in group L. The difference in virus dose in blood meals was clearly observed. Heads were negative at 0 dpf, except for one in groups L and M and two in group H. Likely, contamination by decapitation, virus on mouth parts, or incomplete swallowing of the blood meal can occur. At 10 dpf, positive results of bodies and heads indicated infection, replication and dissemination of wtBTV11. Bodies and heads in all three groups clearly segregated in PCR-positives and negatives. The percentage of PCR positives at 10 dpf was similar for bodies and heads in each group, and was approximately 40%, 79% and 89% for group L, M and H, respectively (Fig. 5). The mean Cq value at 10 dpf for each group mainly differed by the difference in percentage of infected midges, since the maximal Cq value for individual bodies was ±20 and for heads 22–23. Obviously, the difference in mean Cq values between 0 and 10 dpf is less evident for bodies than for heads, in particular for group H. Taken together, infection, replication and dissemination of wtBTV11 by blood-feeding of competent midges is demonstrated. As expected, the efficiency of infection of midges is dose-dependent. The 50% infective virus titer - one 50% Midge Alimentary Infective Dose (MAID_50_) - is roughly calculated to a blood meal titer of ±2 × 10^5^ TCID_50_/ml for wtBTV11. Thus, one MAID_50_ corresponds to ±20 TCID_50_ wtBTV11 in a blood meal estimated to be 100 nl for fully engorged competent *C. sonorensis* midges.

### Differential virus replication in midges by deletion of 72 amino acid (aa) codons in Seg-10

BTV1 deficient for NS3/NS3a expression, named Disabled Infectious Single Animal (DISA) vaccine does not propagate in midges after intrathoracic inoculation [9]. Here, we studied virus propagation in detail after blood-feeding of BTV11(S10^del^) lacking a region in NS3/NS3a encompassing Late Domain, and is called ‘DISA’. Midges were blood-fed containing ±2 × 10^6^ TCID_50_/ml DISA or rgBTV11. This corresponded to ±200 TCID_50_ which is ±10 MAID_50_, and thus sufficient to infect a high percentage of midges. In addition, virus replication was studied after intrathoracic inoculation with the same amount of DISA vaccine virus.

**Fig. 6:**
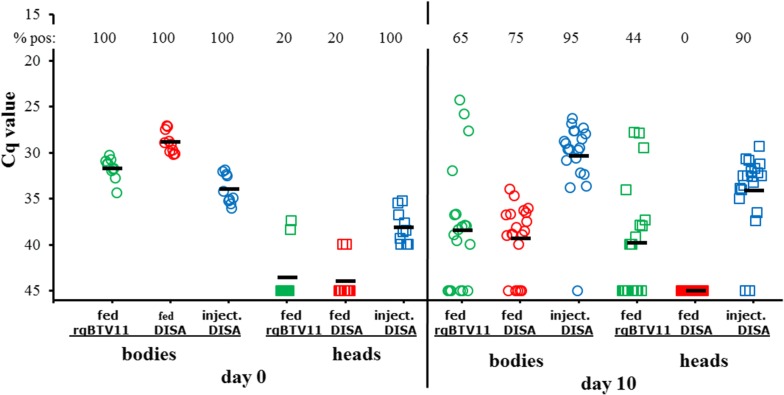
Virus propagation *in vivo* of rgBTV11 and BTV11(S10^del^) in midges. Colonized *C. sonorensi*s were fed with blood containing rgBTV11, or were fed or injected with BTV11(S10^del^). Viral RNA was detected and semi-quantitated by PCR expressed in Cq values for individual bodies (circles) and heads (squares) at day 0 and day 10 after feeding or inoculation. The mean Cq value (bars) and percentage of PCR-positives of each group are indicated

Virus uptake by feeding or inoculation was confirmed by 100% PCR positive bodies at 0 dpf and 0 dpi (Fig. 6). Mean Cq values varied between groups, despite a normalized virus titer of rgBTV11 and DISA. As expected, the majority of heads (8 out of 10 fed midges) were PCR-negative at 0 dpf, whereas inoculation resulted in 100% PCR-positive heads. This suggests that inoculated virus rapidly disperses *via* the haemolymph into the head.

At 10 dpf, 65% of the bodies and 44% of the heads of midges fed with rgBTV11 were PCR-positive, indicating infection, replication and dissemination of rgBTV11 (Fig. 6). However, the percentage of infected midges was lower than expected based on the calculated high dose of 10 MAID_50_. Despite of 75% PCR-positive bodies at 10 dpf with DISA, all heads remained PCR negative. Likely, DISA initially infected gut cells but is not released into the haemolymph and was therefore not disseminated to the head. DISA-propagation in competent midges after oral uptake was unsuccessful by lack of Late Domain in NS3/NS3a protein. After intrathoracic inoculation, a small decrease in mean Cq value (more virus) in bodies and heads was observed at 10 dpi. This indicated infection and replication of DISA after intrathoracic inoculation similarly as observed in bodies after blood-feeding. Deletion of Late Domain of NS3/NS3a protein caused differential virus propagation *in vivo*. We conclude that functional NS3/NS3a is essential for BTV propagation in competent midges after oral uptake. These results confirmed the DISA principle of BTV lacking functional NS3/NS3a expression.

### Differential virus replication in midges by point mutations

**Fig. 7:**
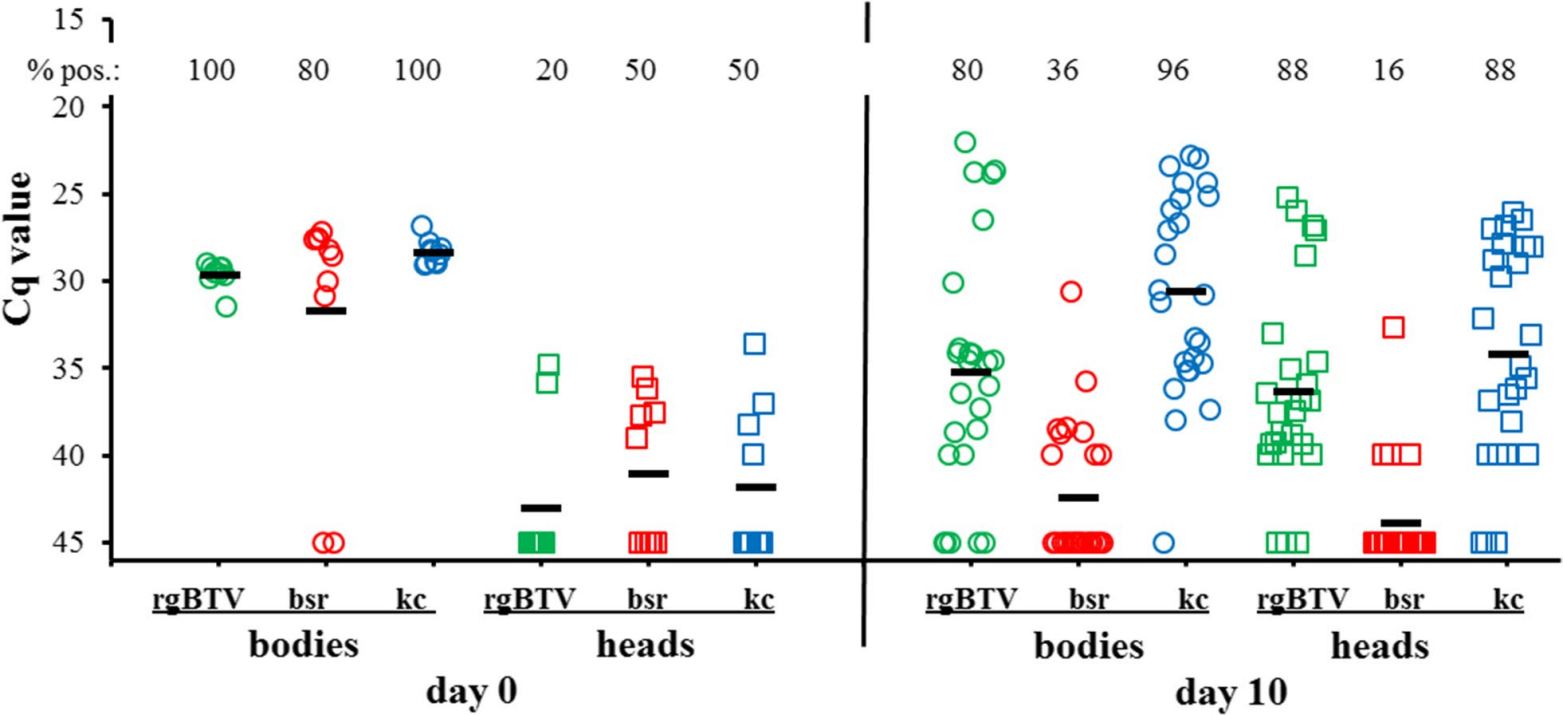
Virus replication *in vivo* of rgBTV11 variants in midges. Colonized *C. sonorensis* were fed with blood containing reverse genetics derived BTV11 (rgBTV), BTV11(S1^26^)bsr (bsr) or BTV11(S1^26^)kc (kc). Viral RNA was detected and semi-quantitated by PCR-testing expressed in Cq values for individual bodies (circles) and heads (squares) at 0 dpf (day 0) and 10 dpf (day 10). The mean Cp values (bars) and percentages of PCR-positives of each group are indicated

BTV11(S1^26^)bsr, BTV11(S1^26^)kc, and rgBTV11 were fed to midges using a normalized virus titer of ±2 × 10^6^ TCID_50_/ml (Fig. 7). Virus uptake was confirmed by PCR-positivity for bodies at 0 dpf for all three groups, except for two fed with BTV11(S1^26^)bsr. As expected, rgBTV11 efficiently propagated in blood-fed midges, since bodies and heads at 10 dpf were 80% and 88% PCR-positive, respectively (Fig. 7). Similarly, BTV11(S1^26^)kc propagated in midges, as indicated by > 90% infected midges at 10 dpf. In contrast, BTV11(S1^26^)bsr showed 36% and 16% PCR-positive bodies and heads at 10 dpf, respectively. This indicated that infection of midges by mammalian cell-adapted BTV11(S1^26^)bsr was less efficient than by *Culicoides*-adapted BTV11(S1^26^)kc and rgBTV11. In addition, mean Cq values were higher (less virus) for BTV11(S1^26^)bsr than for BTV11(S1^26^)kc and rgBTV11 (Fig. 7). Apparently, BTV11(S1^26^)kc propagated similar to, or even slightly better than, rgBTV11 and more importantly, much better than BTV11(S1^26^)bsr. We conclude that infection, replication and dissemination of BTV11(S1^26^)kc is more efficient in competent midges than of BTV11(S1^26^)bsr. Furthermore, the increased propagation *in vitro* and *in vitro* of BTV11(S1^26^)kc is likely caused by one aa mutation in outer shell protein VP2.

## Discussion

Spread of arthropod-borne viruses depends on virus replication in the host and the competent insect vector leading to viremia in the host and virus excretion in insect saliva, respectively. wtBTV11 infects competent *C. sonorensis* midges after oral uptake and can reach the midge head as early as on 3 dpf and definitely on 7 dpf [9]. In this previous study, a very high virus titer of 10^8.2^ TCID_50_/ml wtBTV11 served as control for virus propagation after blood-feeding as indicator for vector competence of midges [9]. A clear segregation of infected and non-infected bodies and heads at 10 dpf further demonstrated the efficiency of infection, replication and dissemination of virus after blood-feeding. In the present study, we determined that ±20 TCID_50_ of wtBTV11-infected 50% of fully engorged midges (Fig. 5), which is named one 50% Midge Alimentary Infective Dose or MAID_50_. One MAID_50_ of wtBTV11 corresponds to a complete blood meal of 100 nl containing ±2 × 10^5^ TCID_50_/ml wtBTV11. Previously, a virus titer of 10^5.8^ TCID_50_/ml (±6 × 10^5^ TCID_50_) of reverse genetics derived BTV1 reassortant (corresponding to ±3 MAID_50_ of wtBTV11) did not infect competent midges, although intrathoracic inoculation was successful [9]. Clearly, infection of midges by blood-feeding depends on the MAID_50_ but likely the used virus strain is even more important. The midge feeding model was used to study propagation of BTV11 mutants in more detail.

BTV11 was rescued by reverse genetics aiming to identify viral genes involved in vector competence. Expectedly, ‘synthetic’ BTV11 (rgBTV11) replicated well in BSR and KC cells (Fig. 1), and infected *C. sonorensis* midges after blood-feeding (Figs. 6 and 7). However, mean Cq values were higher (less rgBTV11) than for wtBTV11, but PCR results for bodies and heads at 10 dpf varied more for rgBTV11-infected midges (Fig. 5 *versus* Figs. 6 and 7). This variation suggests that infection, replication or dissemination of rgBTV11 is slightly slower than of wtBTV11. However, the percentage of infected midges differed between feeding experiments with rgBTV11 suggesting some variation in competence between hatches/batches of midges (Figs. 6 and 7). Nonetheless, rgBTV11-infected midges after oral uptake, was released into the haemolymph, and was disseminated to the head. Thus, rgBTV11 is a suitable virus backbone to test BTV11 mutants in the midge feeding model.

NS3/NS3a protein is the prototype BTV gene involved in differentiating virus replication in cell lines of the mammalian host and the midge vector [10, 11, 35]. NS3/NS3a knockout mutants have been developed as DISA vaccines [36]), and show ‘differential virus replication’ *in vivo* [9]. BTV11(S10^del^) (here named DISA) lacking Late Domain of NS3/NS3a demonstrates similar characteristics (Figs. 1, 6). Here, we also showed that blood-feeding with a high titer of DISA vaccine virus infected midguts and weakly replicated but was not disseminated to the head (Fig. 6). In conclusion, DISA vaccine virus with a small deletion of 72 aa codons in NS3/NS3a protein cannot reach salivary glands and will not be excreted by midges to susceptible hosts during blood-feeding. The deletion in BTV11(S10^del^) corresponds to the in-frame deletion of 77 aa codons in the experimental DISA vaccine for African Horse Sickness [36, 37]. Similar to the BTV DISA vaccine, this NS3/NS3a mutant of African horse sickness virus (AHSV) is not virulent, and showed similar *in vitro* characteristics. It is likely that it would also be blocked in virus release and dissemination in blood-fed midges. Taken together, a small deletion in NS3/NS3a protein encompassing Late Domain is sufficient to establish nonvirulence, the DIVA principle (Differentiating Infected from Vaccinated), and the DISA principle (Disabled Infectious Single Animal) (reviewed in [38]).

Pullinger et al. [20] have shown differential virus replication *in vitro* for several reassortants of typical BTV1 and atypical BTV26. In this study, we aimed to determine differential virus replication *in vivo*, and rescued typical BTV11 with S1^26^. Rescue of BTV11(S1^26^) was less efficient than BTV11, and adaptive changes after rescue were assumed to improve virus growth on BSR cells. In agreement with Pullinger et al. [20], BTV11(S1^26^) initially showed limited virus production in KC cells (Fig. 3). Interestingly, we were able to adapt BTV11(S1^26^) to KC cells. BTV11(S1^26^)kc had obvious phenotypical differences as observed by increased virus growth and enlargement of immunostained foci in infected KC cell monolayers (Figs. 2, 4). Additionally, replication of virus passaged rgBTV11kc was slightly increased in KC cells and immunostained foci in KC cell monolayers were also slightly larger compared to virus passaged BTV11bsr. These results indicated adaptation for virus growth in KC cells for both BTV11(S1^26^)kc and rgBTV11kc. BTV11(S1^26^) was independently rescued again, and in addition, an S1^26^ mono-reassortant of BTV1 strain RSArrrr/01 was rescued (BTV1(S1^26^) according to previous results [20]. As expected, both S1^26^ mono-reassortants showed hardly replication in KC cells but could be adapted to KC cells (Fig. 2). We conclude that differential virus replication of S1^26^ mono-reassortant of BTV1 and BTV11 was abolished by virus passages in KC cells suggesting adaptation mutations in one or more genome segments.

Adaptation mutations were identified but, more importantly, corrections of chimeric interactions and adaptation mutations could be distinguished (Table 1). BTV11kc contained one aa mutation in VP2 protein, whereas BTV11bsr did not contain mutations. Similarly, rescued virulent BTV8 and nonvirulent BTV6 did not change after rescue in BSR cells [25]. Of note, passaged BTV11kc and BTV11 bsr were partially sequenced and mutations outside the regions of interest cannot be ruled out (Table 1). Viruses exhibit adaptation mutations known as genetic drifting, but arise of adaptive changes strongly depends on the selection pressure in the field or during *in vitro* virus passages [39–42]).

Five nucleotide mutations were found in rescued BTV(S1^26^) (Table 1). Four mutations implicated aa mutations in VP1^26^, VP4^11^ and two in NS2^11^. The aa mutations were rapidly selected after virus rescue assuming interactions with exchanged VP1^26^ and suggested a strong selection. Likely, these improve chimeric interactions between VP1^26^ and BTV11 proteins since these were not found in BTV11. In addition, no aa mutations were found in S1^26^ after passages of BTV26 on BSR cells. VP1, VP4, and NS2 are all associated with the replication machinery. Amino acid mutation D309G in VP1^26^ is not unique and seems to be associated with eastern topotypes of BTV. BTV1(S1^26^) contained the same point mutation leading to the D309G aa mutation, while independently rescued BTV11(S1^26^) had a point mutation G936A leading to aa mutation D309N. These results demonstrate the importance of mutating Asp on aa position 309. Asp-309 is mapped in the unmodelled region between NTD and PD of RdRp and has been suggested to interact with virus proteins of the replication complex [29]. We propose that D309G or D309N improves chimeric protein interactions of VP1^26^ with proteins of BTV1 or BTV11 resulting in increased virus replication in both BSR and KC cells (Fig. 4).

The aa mutation L399S in VP4^11^ of BTV11(S1^26^) is a non-conserved aa residue. The VP4, a capping enzyme, is part of the replication complex and catalyses the formation of a cap1 structure at the 5’ termini of core RNA transcripts. VP4 has distinct domains for its different activities [43]. The aa mutation L399S is located in the second region of the N7MTase domain (residues 110–170 and 380–500) suggesting a role in this activity. However, it is more likely that the L399S aa mutation improves chimeric interactions between VP4 and VP1 than capping activities of VP4, since assembly of the replication complex starts with interactions between VP1 and VP4.

NS2^11^ of rescued BTV11(S1^26^) contained aa mutations L171F and E221G. NS2 of atypical BTV25-27 also contained F-171, and E-221 in NS2 of atypical BTVs is highly conserved within a variable region. Independently rescued BTV11(S1^26^) also contained L171F but E221G was not found, whereas S8^1^ of rescued BTV1(S1^26^) was not mutated. We speculate that these aa residues of NS2 are involved in interactions with VP1. NS2 recruits viral ssRNA from the cytoplasm, but its interactions with core proteins VP1, 3, 4, 6, and 7 are largely unknown (reviewed in [44]). Our results support the biological evidence that NS2 is associated to VP1.

One aa mutation in VP1^26^, one aa mutation in VP3^11^, and one silent mutation in S1^26^ were found in BTV11(S1^26^)kc (Table 1). These mutations were either still mixed, like the silent mutation on nucleotide position 2393 in S2^11^, or arose very late after several virus passages. We assume that selection for these mutations was very weak or absent and consider these as natural variations after rescue of (clonal) virus. More importantly, no obvious mutations in VP1^26^ were found that are associated to adaptation to virus growth in KC cells. Surprisingly, we found that VP1^26^ is not involved in differential virus replication *in vitro*.

The most obvious difference between BTV11(S1^26^)kc and BTV11(S1^26^)bsr is nucleotide mutation A981G in S2^11^ resulting in E321G in VP2^11^ (Table 1). BTV11kc contained the same aa mutation and is the only difference compared to BTV11bsr. Apparently, aa mutation E321G in VP2 favoured virus replication *in vitro* (Fig. 4). Independently rescued BTV11(S1^26^) contained also mutations in VP2^11^; E401G and I503V, and the S1^26^ chimeric virus based on BTV serotype 1 also contained one N229S mutation in VP2^1^. In a previous study, BTV8/net07/e1 was also passaged in KC cells (BTV8/net07/e1kc3) leading to two nucleotide changes of which one resulted in R400G in VP2^8^ [25]. VP2 reportedly binds to the cell surface receptor and to a cell surface glycoprotein by its sialic acid binding domain in the central hub domain of VP2 [45]. We noticed that adaptive mutations in VP2^11^ are located in or close to the externally exposed flexible tip domain of VP2 mapped to aa 191–407. A similar region of VP2 (aa residues 278–504) has been shown for AHSV [46]. This region in AHSV-VP2 is not essential for *in vitro* virus replication in both cell types, but the corresponding region (aa residues 284–510) in AHSV4-VP2 seems to be involved specifically in virus replication in KC cells, since virus release from KC cells of this AHSV deletion mutant was slightly delayed [47]. Altogether, mutations in VP2 of these midge-borne orbiviruses are strongly associated with adaptation to KC cells confirming that VP2 is involved in differential virus replication *in vitro*.

In order to identify proteins or domains involved in differential virus replication *in vivo* and thus important for vector competence, RdRp VP1 was a promising candidate [20]. However, no domain in VP1 involved in differential virus replication was identified. Instead, VP2 was found to be a key candidate affecting vector competence. Indeed, outer shell proteins VP2 and VP5 of atypical BTV26 also blocked virus replication in KC cells [20]. Differential virus replication *in vitro* caused by one aa mutation in VP2 was found in rgBTV11 but was more pronounced in combination with VP1 of atypical BTV26 (Fig. 4). More importantly, BTV11(S1^26^)kc propagated better in competent midges than BTV11(S1^26^)bsr (Fig. 7). It is very tempting to speculate that aa mutation E321G in VP2^11^ solely caused this differential virus replication *in vivo*. Indeed, partial sequencing of viral RNA isolated from fed midges at 10 dpf confirmed previous mutations, indicating no reversion or selection of certain mutations after oral uptake. More research on VP2 is needed on independently rescued and adapted virus variants to study the role of VP2 in vector competence.

BTV8 re-emerged in France in 2015 and reportedly spreads much slower than the BTV8 strain causing the huge epidemic in north-western Europe in 2006–2009. These BTV8 strains are closely related and contain 11 aa differences scattered over seven genome segments/proteins, including three in VP1 and one in VP2 [48]. The re-emerging BTV8 strain is less virulent, causing a lower viremia, and showing a reduced vector competence [48]. The latter might be caused by the lower viremia in the host, and reduced virus propagation in midges after standardized blood-feeding cannot be excluded. Reverse genetics for BTV8 and the midge feeding model presented here with separate testing of bodies and heads, could be used to elucidate the role of each of these aa mutations in virus propagation in midges.

## Conclusions

The midge feeding model, including decapitation and separate testing of individual bodies and heads, is a suitable approach to identify viral factors involved in propagation of virus mutants in more detail. One MAID_50_ (50% Midge Alimentary Infective Dose) of wtBTV11 infects 50% of fully engorged midges and corresponds to a complete blood meal of 100 nl containing ±2 × 10^5^ TCID_50_/ml or 20 TCID_50_ wtBTV11. Clearly, infection of midges by blood-feeding depends on virus uptake but the used virus strain is even more important. A small 72 amino acid in-frame deletion of NS3/NS3a protein completely blocks virus dissemination in blood-fed midges. Further, detailed knowledge of protein-protein interactions in the virion was generated by analysis of BTV reassortants. In addition, a point mutation in outer shell protein VP2 was identified in *Culicoides*-adapted BTV that is associated with differential virus replication *in vitro* and *in vivo* and thus with vector competence. In conclusion, two examples of small changes in BTV are shown which strongly affect virus infection, replication and dissemination of virus in competent midges. All these processes are part of the key mechanism crucial for vector competence and thus for spread of the bluetongue virus.

## Data Availability

Data supporting the conclusions of this article are included within the article. The datasets used and/or analysed during the present study are available from the corresponding author upon reasonable request.

## Abbreviations

BT: Bluetongue
BTV: bluetongue virus
CPE: cytopathogenic effect
Cq: quantification cycle
CTD: C-terminal domain
DMEM: Dulbecco’s modified Eagle’s medium
dpf: days post-feeding
dpi: days post-inoculation
FBS: foetal bovine serum
hpi: hours post-infection
IPMA: immunoperoxidase monolayer assay
MAID: midge alimentary infective dose
MOI: multiplicity of infection
NTD: N-terminal domain
PCR: polymerase chain reaction
PBS: phosphate-buffered saline
PD: polymerase domain
RdRp: RNA-dependent RNA polymerase
rg: reverse genetics
TCID: tissue cell infective dose
WBVR: Wageningen Bioveterinary Research
wt: wild type
